# Becoming diplomats with boundaries - a thematic analysis of relatives’ experiences with group-based psychoeducation about bipolar disorder

**DOI:** 10.1186/s12888-025-07219-y

**Published:** 2025-09-01

**Authors:** Julie Ravneberg Stokholm, Anna Kristine Waldemar, Lars Vedel Kessing

**Affiliations:** 1 Psychiatric Center Copenhagen, The Copenhagen Affective Disorder Research Center (CADIC), Hovedvejen 17, 1. floor, 2000 Copenhagen, Denmark; 2 Department of Clinical Medicine, Faculty of Health and Medical Sciences, University of Copenhagen, Copenhagen, Denmark; 3 Competence Centre for Recovery, Mental Health Services in the Capital Region of Denmark, Copenhagen, Denmark

**Keywords:** Group-based psychoeducation, Bipolar disorder, Relatives, Caregivers, Family, Partner, Parents, Qualitative thematic analysis

## Abstract

**Background:**

Group-based psychoeducation for relatives of patients with bipolar disorder (BD) is offered at the Copenhagen Affective Disorder Clinic, Denmark. This qualitative study explores relatives’ experiences with the program, how it influenced them, and key factors shaping these effects.

**Methods:**

We interviewed 10 relatives (5 parents and 5 partners) who had recently completed the psychoeducation program. We included only parents and partners, as they make up 90% of attendees at the clinic’s psychoeducation. The interviews were individual, semi-structured, audio-recorded, transcribed, and analyzed using reflexive thematic analysis. Through a concurrent parallel RCT study involving 206 relatives, we obtained 70 written commentaries from an evaluation questionnaire about the education.

**Results:**

Through the analysis, we developed five themes: A: *Relating through shared experiences*, B: *Understanding the land of Bipolar Disorder*, C: *Trusting the Treatment Means Sharing the Burden*, D: *Setting boundaries to protect yourself*, and E: *Becoming a diplomat*. Participants valued recognizing themselves in others, but meaningful recognition required similarity in relation (partner or parent) and BD type I or II (theme A). Understanding BD and its treatment was crucial for participants (B & C), with some participants reporting significant knowledge gains while others were already familiar. Some participants found great relief in meeting the clinicians and learning to trust the treatment, as they had previously been concerned about its quality and felt a self-imposed responsibility (theme C). Many, especially partners, found setting boundaries challenging and wanted more focus on this subject in the education (D). In theme E, we explore how relatives reflected on their ability to cope in difficult situations by being diplomatic and strategic and drawing on the insights described in themes A–D.

**Conclusions:**

Group-based psychoeducation for relatives of patients with BD was feasible and addressed core support needs, but adjustments could enhance its impact. In a large-group setting, participants could be divided into smaller discussion groups based on their relationship to the patient and/or the patient’s BD type. Additionally, we propose incorporating ‘diplomats with boundaries’ as a positive caregiving model, conceptualized through our analysis.

**Trial registration:**

The investigation was conducted as part of the R-Bipolar study registered at ClinicalTrials.gov, ID NCT06176001, 19/12/2023. Submission for ethical approval was not required (Journal-nr.: 21063013).

**Supplementary Information:**

The online version contains supplementary material available at 10.1186/s12888-025-07219-y.

## Background

Bipolar disorder (BD) is a severe mental illness characterized by an early age of onset and a life-long course with a high risk of recurrence of manic and depressive episodes [1], and a severe impact on functioning and quality of life [2, 3]. The episodic nature of BD and the diverse symptoms in depressive, euthymic, hypomanic, manic, and mixed episodes, contribute to the burden for the close relatives [4]. Even when the patient is euthymic (between phases), relatives still experience distress related to the patient’s problem behavior, e.g. mood instability, activity instability, irritability, and withdrawal [5, 6].

Modern psychiatry is primarily based on outpatient services. Admission to a psychiatric ward is reserved for severe or acute conditions, e.g. psychosis or high risk of suicide. Patients with BD are therefore at home also when they experience difficult depressive, hypomanic, or manic episodes. Thus, modern psychiatry is to a high degree dependent on the work of relatives [7].

Relatives can play a significant role in supporting patients emotionally, and practically, and in patient’s adherence to treatment. For example, relatives can help observe prodromal symptoms of upcoming mood episodes and therefore be important for successful early interventions [8, 9]. On the other hand, a family environment characterized by critical comments, hostility, and emotional over-involvement can adversely affect the course of BD [10, 11].

A recent Danish nationwide study found that a high degree of caregiver-reported involvement was statistically significantly associated with high patient-reported improvement and overall satisfaction with care [12]. Psychoeducation of relatives of persons with BD has been recommended in the NICE guideline from 2014 [13] and several studies since [10, 14–16]. At the Copenhagen Affective Disorder Clinic, Mental Health Services, Capital Region of Denmark [17] we offer group-based psychoeducation for relatives. The psychoeducation consists of six weekly sessions each two hours, in groups of 20–35 relatives.

The need for a better understanding of the mediating and moderating mechanisms in family interventions is emphasized in two systematic reviews on the effects of family interventions [10, 18]. Qualitative analysis can help identify both mediating and moderating mechanisms as well as feasibility, acceptability, concerns, and unintended consequences of an intervention [19–21]. Traditionally in randomized controlled trials, the generalizability of findings is based on differences in characteristics of the settings and populations. But preferentially, an assessment of generalizability should also include an understanding of why, when, and how an intervention works [22, 23].

This qualitative study aims to get insights into the *relatives’ experience of the group-based psychoeducation program*,* how it influenced them*,* and identify the factors that appeared to contribute to these effects and experiences.*

This study is part of the R-bipolar study which combines qualitative insights with a randomized controlled trial (RCT) on the effectiveness of group-based psychoeducation for relatives of patients with bipolar disorder [24].

## Methods

In this paper, we adhered to *the APA Publications and Communications Board task force report on reporting standards for qualitative primary*,* qualitative meta-analytic*,* and mixed methods research in psychology* [25].

### Description of the setting and intervention

The Copenhagen Affective Disorder Clinic is a specialized outpatient unit within Denmark’s Public Mental Health Services, Capital Region. It provides treatment services for approximately 350 patients newly diagnosed with BD annually. The clinic comprises approximately 20 experienced clinicians, including 7 specialists in psychiatry and 7–8 nurses. Treatment consists of evidence-based medical treatment and group-based psychoeducation for patients according to international guidelines [26, 27].

In addition, the clinic has since 2012 offered group-based psychoeducation for relatives. The initiative was inspired by results from Barcelona [28] and observations in the clinic that the relatives are both very burdened and important for the course of BD. Preliminary data suggest that group-based psychoeducation for caregivers may have beneficial effects, but larger and more well-designed trials are needed before clinical recommendations can be made with confidence [18].

Group-based psychoeducation in the Copenhagen Affective Disorder Clinic for relatives of patients with BD consists of six sessions each of two hours and led by experienced clinicians: a physician and a nurse. In one of the sessions, a guest teacher shares his personal experiences with having bipolar disorder. The groups are relatively large with 20–35 participants, which increases feasibility in clinical settings and decreases costs. In terms of the content, the primary focus is to provide a comprehensive understanding of bipolar disorder, including its definition, available medical and psychotherapeutic treatments, and the common challenges that individuals with the disorder face in their daily lives. The final session concentrates on the relative’s role, providing insights into common responses and difficulties faced by relatives, along with suggestions on how you can support your loved ones. Additionally, it emphasizes the significance of self-care. The psychoeducation is not presented or intended as group therapy but includes therapeutic elements.

The patients with BD are offered group-based psychoeducation as part of their treatment plan and do not attend the psychoeducation for relatives. Patients can invite 1–2 relatives to participate in the psychoeducation program for relatives. The patients are recommended to enroll their relatives for psychoeducation after they have started in their own psychoeducation group. This is to avoid situations where the patients feel overtaken if the relatives know more about BD than they do themselves. The attending relatives are usually parents or partners, but patients are also welcome to invite a friend, sibling, adult child, etc.

The program for relatives has consistently received positive feedback and has maintained high attendance rates, suggesting its relevance in meeting the needs of the community. The feasibility of the program is generally considered high as it has been viable in real-life settings for more than 10 years with low costs per participant.

### Research design and theoretical approach to inquiry

JRS conducted 10 semi-structured interviews with participants who had finished the group-based psychoeducation course for relatives of patients with bipolar disorder. The interviewees also participated in the RCT earlier described [24]. The interviews were audio-recorded, transcribed, and analyzed using reflexive thematic analysis [29]. In addition to the interview data, we included the free-text answers from an evaluative questionnaire that 206 participants from the R-bipolar RCT were invited to complete. The evaluative questionnaires were not used to develop the analysis, but rather to support it and give voice to participants from a bigger sample.

In our analysis, we employed an experiential orientation, focusing on the participants’ individual experiences and personal interpretations. In continuation of our experiential orientation, we adhere to a critical realist ontology and an understanding of language as a relatively straightforward tool for communication [29](p. 169). We did not a priori decide on a specific ‘explanatory’ theory to underpin our analysis. Rather, we approached the analysis inductively and themes were not predefined by theory.

### Rationale for the selected design

We chose individual interviews as our primary data source to capture the participants’ personal experiences without the potential influence of group dynamics. Conducting individual interviews also reflects that the participants are individuals in their roles as relatives, thus the group’s understanding of the phenomena is not central.

As our analytical approach, we chose reflexive thematic analysis as presented by Braun and Clarke [30] and relied on their recent book, “Thematic Analysis – A Practical Guide” from 2022 [29], as a valuable resource and reference.

Reflexive TA is a good fit for our study because it offers an accessible method to develop and interpret meanings across our dataset resulting in central themes. Another advantage is that TA can be combined with different theoretical stances. The notion of a *reflexive* approach to TA implies that the researcher critically reflects on his or her role in the process [29] (p12).

Before the study, we planned to conduct ten interviews, with the option of including more if needed. After conducting the ten interviews, we were satisfied with the richness of the data and chose not to conduct additional interviews. This is in line with our analytical approach reflexive thematic analysis as Braun and Clarke argue that the researchers should reflect on whether the dataset is “rich” and meets the study’s goals rather than an arbitrary “data saturation” is achieved [29, 31].

We wanted to purposefully select participants who were parents or partners as they are the typical attendants for the psychoeducation. If we had included other types of relatives (e.g. friends) it would easily skew the results in a small sample and would potentially give less useful results, as the themes might come out more divergent and transferability would be less obvious. Additionally, we aimed to achieve an equal distribution between the sexes.

### Study participants

Table 1 presents interview participants with their relation to a patient. Anonymization is achieved through pseudonyms and modifications in their ages.

**Table 1:** Interview participants

Pseudonym	Relation to the patient	Age
**Line**	Spouse	35
**Nanna**	Mother	56
**Camilla**	Partner	27
**Mads**	Spouse	31
**Tanja**	Mother	60
**Bent**	Father	62
**Anders**	Partner	22
**Morten**	Partner	33
**Lasse**	Father	58
**Sofie**	Mother	57

When we use free-text answers from the survey, the same information will be presented under the specific quote.

### Researcher description and reflection

In line with our choice of reflexive thematic analysis, we recognize that our positions as researchers shape all stages of the study, from data generation to analysis. Below we will describe and reflect on the position from which we approached this study. In terms of personal reflexivity JRS has personal experience with being a close relative to a person with depression, but not with bipolar disorder. JRS is therefore not an “insider researcher” [32] and did not actively use her own experiences in the interviews or analysis.

JRS and LVK are medical doctors and AKW registered nurse. As healthcare professionals, this background gives us knowledge about psychiatric diseases and an understanding of the healthcare system. However, our professional background may also mean that we are accustomed to dominant clinical logics and practices, which we might be less inclined to question or view critically. This potential bias was addressed in the research group by continuously discussing possible different perspectives and interpretations of the data including any underlying assumptions in the interview and analysis process.

The design and orientation of the qualitative study also reflect that it was conducted in parallel with a randomized controlled trial (RCT). We aimed for the two studies to be in dialogue with each other, and to ensure that our findings would be directly useful in clinical practice. To support this aim, we adopted an evaluative focus and drew on an experiental approach, which we considered ontologically and epistemologically compatible with the realist assumptions underpinning the RCT.

### Participant recruitment and selection

To recruit, JRS showed up in the final session of group psychoeducation for relatives of BD in June 2022 and invited relatives to participate in the qualitative study. This strategy brought the first eight participants to sign up on the same day. A few days later JRS followed up by telephoning two male relatives who had attended the psychoeducation and invited them to participate, which they accepted. This was done to ensure ten participants and an even distribution between the sexes. The participants had previously met JRS when they were included in the R-bipolar RCT approximately 4 months before.

The other data source in the form of free-text evaluations was retrieved from an online questionnaire that was sent to 206 participants in the RCT after they had completed the psychoeducation program. In the evaluation questionnaire, the respondents had the option to elaborate on their evaluation in a short free-text format, thus they could comment on anything concerning the education.

### Data collection

For the interviews JRS, AKW, and LVK developed the interview guide with open-ended questions that approximated the aim of the study: *to get insights into the relatives’ experience of the psychoeducation program*,* how it influenced them and identify the factors that appeared to contribute to these effects and experiences.*

JRS performed a pilot-interview which AKW observed. After this pilot interview, we adjusted the elaborating questions in the interview guide (see Appendix 1). JRS conducted the remaining interviews within 9 weeks after they had completed the psychoeducation course. The timing was flexible to accommodate the participants’ schedules.

The interviews took place at JRS’s office in the psychiatric unit’s research department. Each interview lasted approximately one hour. A reflective notebook was kept by JRS doing the process of both data collection and analysis, in addition to summaries from meetings with LVK and AKW and their written feedback.

### Data analysis strategy

Here follows a description of the process through the six analytical phases of reflexive TA [29]:

- Phase 1: Familiarize yourself with the data. The interviews were transcribed by a psychologist student assistant. JRS read the transcripts and listened to the audio files to get familiar with the data.
- Phase 2: Coding. JRS coded directly in the QACDAS tool Nvivo14. During the process of coding JRS revised and collated codes on an ongoing basis. JRS and AKW met and discussed the coding of a couple of interviews, as well as possible initial themes in phase 3.
- Phase 3: Generating Initial themes. JRS categorized the initial codes that (a) seemed to fit together around a common subject and (b) were relevant to the research question. This exercise of generating initial themes also gave rise to a need to go back to phase 2 for refinement and collating of some codes.
- Phase 4: Developing and Reviewing Themes. JRS tested the ‘fit’ of the themes by going through the coded extracts for each theme and re-reading the whole dataset. JRS deliberately read the interviews in a new sequence, as the order might influence the understanding of the data. This exercise of re-reading the data resulted in fine coding of the data and inspiration for collapsing a couple of themes, but the core of the developed themes and the framework seemed to have a good fit with the data.
- Phase 5: Refining defining and naming themes. JRS evaluated if each theme was demarcated and had a strong core concept. This was done in the process of writing a brief synopsis of each theme and deciding on names for each theme. Refining the themes was an iterative process that continued through the review process of the article.
- Phase 6: Writing up. The process of writing was an integrated part of the development of the analysis, and JRS continuously involved LVK and AKW in the writing process.

## Results

The analysis resulted in five themes. The themes are presented in Table 2, with a short description:

**Table 2:** Theme summaries

Theme name	Description
**A) Relating through shared experiences**	It was rewarding to meet, relate, and reflect with other relatives in similar circumstances. However, being in ‘similar circumstances’ where only experienced when speaking with someone who shared the same type of relationship (partner or parent) to the person with BD.
**B) Understanding the land of Bipolar Disorder**	The psychoeducation offered a good understanding of BD which was deemed crucial for effective communication with the person living with BD and for navigating life alongside the condition. However, some relatives felt that the psychoeducation was tailored to a ‘beginner level,’ leaving more advanced needs unaddressed.
**C) Trusting the Treatment Means Sharing the Burden.**	Through the education, the relatives developed trust in the treatment plan, the clinicians, and the medication. This brought a sense of relief, as some had previously worried that the treatment might not be adequate, making it hard to confidently entrust the clinic with responsibility for the care.
**D) Setting Boundaries to protect yourself**	The relatives emphasized the importance of ‘setting boundaries’ on how much BD should define their everyday life and relationships. However, they found it very challenging to establish these boundaries and suggested that the topic should receive more attention in the education
**E) Becoming a diplomat**	The relatives expressed that developing good diplomatic skills, such as conflict management, communication, and timing, was important for the relation to the person with BD and their everyday lives. Some participants felt that this topic should have been explored in more depth.

In the following, we will present each theme and underpin them with data. We will focus primarily on the data from the qualitative interviews on which the analysis was based. Secondly, we will add insights from the written comment section of the questionnaire to show how the themes applied to a broader group of relatives. A total of 70 out of 206 participants in the RCT chose to write a comment. It is worth noting that the comment section was “free-text” thus reflecting what the respondent *chose* to comment on, without being prompted.

### Theme A: Relating through shared experiences

The participants emphasized the positive aspects of meeting other relatives of patients with BD.

Recognizing themselves in others, helped the relatives to feel that they were not alone and to exchange experiences, which is exemplified in the following quote:

*“Everything they [the other relatives]*,* said*,* well*,* I could have said the same myself*,* and it helped me a lot. Because I often think*,* ‘Am I the only one?’ I know I’m not*,* but sometimes you get that feeling of being very alone. And that no one understands*,* not even professionals*,* not family members or friends. So I thought that was really nice. And not so much those who were parents. I couldn’t really relate to them*,* but I could relate to those who had them as partners.”*

*Line*,* spouse*,* age 35*.

In this quote, Line mentions that her positive experience of relating with others required that the other person also be a partner to a person with BD. The importance of a specific match with the other person’s situation was recurring throughout the dataset, even though the subject was not prompted in the interview guide. The participants primarily highlighted the differences in the situations and needs of parents versus partners. Additionally, they pointed to the role of BD type (1 or 2) and whether one was in a crisis or working on establishing daily routines. The differences not only affected the relatability *between* the participants but also the needs and expectations of the psychoeducation, the latter will be further elaborated on in theme B.

To sum up the differences that were described between partners and parents: partners typically lived with the person with BD, so they were highly exposed to their partner’s symptoms, and reduced function in everyday life activities. Also, the partners choose to invest their future in the person with BD, and might also have children with them. The following quote from a partner clearly describes the differences experienced:

*“So*,* as parents*,* it’s maybe a question of how much support can I provide*,* and what will my son or daughter allow? (…) As a partner*,* it’s fundamentally different. It’s like*,* should I be in this relationship*,* is it good for me as a person*,* should I step out of it*,* and is there someone else I should be with instead? Or if you’re a partner and have a child with them*,* it’s still the same*,* like what burdens does my partner take versus what do I take in everyday life? (…) And when is there room for me in the relationship? And what about the things I’m struggling with?”*

Morten, partner, age 33.

On the other hand, parents could feel frustrated and powerless being left on the sideline. As their child is an adult and typically does not live at home, the parents lacked information on how their child was doing and how the treatment in the clinic was planned. They might deal with feeling guilt and sorrow over the diagnosis, and confusion on how to best help their adult child.

*“Yeah*,* she’s 26 and lives on her own and all that*,* so I don’t have daily contact with her*,* and in that way*,* I can’t see and fully keep track of how she’s really doing.”*

Nanna, mother, age 56.

The theme of relating with others through shared experiences was strongly reflected in the questionnaire comments. Out of a total of 70 comments, 35 requested more time for dialogue between participants and/or suggested that group discussions be divided based on their relationship to the person with BD.

### Theme B: Understanding the land of bipolar disorder

The name of this theme is inspired by the fact that the relatives were trying to understand something foreign to them, like a new land, and they used words like learn a language, to navigate, recognize patterns etc. In the following, we present data on how a deeper understanding of BD gave the relatives much-needed predictability, helped them to better distinguish between personality and BD, and better communication with the person with BD. Finally, we show how some of the participants were frustrated that they did not learn much new.

In the following quote from a father, he expresses how the psychoeducation helped improve his relationship with his daughter, and how she appreciates his newfound understanding of ‘her land’:

*“Well*,* yeah*,* we’re speaking the same language and have the same understanding [after psychoeducation]. So yeah*,* she feels really good that there’s someone in the family now where we*,* well*,* are on the same page.”*

Bent, father, age 62.

Another father described how this new understanding had made the cyclic nature of BD more predictable to him, and thereby more manageable:

*“Now that we’ve been through this program*,* where we’ve had these 6 sessions of education*,* we can see more clearly*,* more frequently*,* that yes*,* it’s… okay*,* so we’re at that point in the process. Now*,* within the next 14 days*,* something like this will probably happen. And then we’ve been able to kind of keep track of his patterns*,* if you can put it that way*,* right? His cycle.”*

Lasse, father, 58 years.

Besides getting a better idea of what BD is, the psychoeducation was also an opportunity to learn what BD is not. The increased factual knowledge and listening to other peoples’ stories seemed to improve the participant’s pattern recognition and thereby better distinguish between what was BD and what was personality:

*“(…) You could be in a situation where you see something*,* and you wonder*,* is this a symptom of illness*,* or is it just her? And then you see or hear another person (a relative participating in the education) talking*,* and you can say okay*,* that sounds like something they might have in common*,* right?”*

Anders, partner, age 22.

Some of the participants felt they knew most of what was taught during the psychoeducation. There seemed to be a pattern, where the partners had a higher level of knowledge before the psychoeducation than the parents, which influenced their experience. The question of a good match *between* relatives in theme A, thus seemed to be paralleled in the match between the educational material and the participants.

*“(…) it felt like you were teaching people who didn’t really have an idea of what bipolar disorder was. It was kind of like*,* you know*,* a beginner’s course.”*

Anders, partner, age 22.

As the patients typically have been in treatment at the clinic for about a year before the relatives are invited to psychoeducation, the timing also affected the benefit of the education. Of the 70 participants who chose to leave a comment in the questionnaire, 9 of them commented that the psychoeducation was too basic and/or should have been provided at an earlier stage, as this comment exemplifies:

*“The course would have been much more beneficial if it had been earlier in the process – you really need support/knowledge at the beginning*,* when the ‘patient’ is struggling the most.”*

*Written commentary from a mother*,* age 58.*

### Theme C: Trusting the treatment means sharing the burden

The psychoeducation included information about the medical treatment and how the 2-year treatment plan was structured at the clinic. Participants expressed that this knowledge helped them to trust the clinicians, the medical treatment, and how the treatment plan was organized.

A mother pointed out that the fact that the psychoeducation was taught at the clinic and by the clinicians made it very trustworthy:

*“It was persons who actually work specifically with people who have bipolar disorder. I found that very credible*,* especially here [at the clinic]. I mean*,* it was persons who walk the hallways (…) It felt good.”*

Nanna, mother, age 56.

An understanding and overview of the treatment could otherwise be difficult to get when you are on the “sideline” as a parent, as this participating father describes:

*“Well*,* it was just difficult as a relative to get an overview of what steps there are (in the treatment process)? (…) It was getting better and better with the medication*,* and then suddenly she switched to something else. And in the startup phase*,* there were possibilities for some serious side effects. What the hell was that (…)?”*

Bent, father, age 62.

In the following quote, a mother explains how she had been worried that the treatment offered at the clinic was not good enough. In the interview, she described how this worry had made her critically question the treatment which again had led to conflicts with her daughter. The psychoeducation helped her to trust the treatment, which she described as an easing of the relationship:

*“She [the daughter with BD] hasn’t been very forthcoming in telling us what’s going on in here (at the clinic). (…) We haven’t gotten a sense that there was some sort of plan. She just came here for her appointments. But now we come here*,* and then we hear*,* well*,* there’s actually a really good plan throughout this whole process. And it might have removed some frustration and uncertainty from our side if we had known.”*

Nanna, mother, age 56.

Besides easing the relationship with the patient, trust in the treatment could also relieve the relatives from the feeling of being responsible for the treatment, as is indicated by the following quote:

*“I thought like*,* ‘Oh*,* what are they pumping into him? Is this just something they use to drug the patients?’ That [the education] made me relax a bit more about it*,* so I haven’t really thought about it since.*

*(…) the psychiatrist knows about that [the medication]*,* and it has actually been*,* you know*,* where you say: ‘Oh*,* it’s just wonderful that there’s someone who can take responsibility for it.‘“*.

Line, partner, age 35.

The theme of trusting the treatment was not directly commented on in the written commentaries from the questionnaire.

### Theme D: Setting boundaries to protect yourself

The importance of setting up boundaries was highlighted many times by the participants, especially among the partners. These could be boundaries on how much BD should be “allowed” to dominate in the relationship, and how to protect yourself as a relative.

These are questions that inherently lack clear and definitive answers, but as the following quote exemplifies the participants appreciated the opportunity to exchange experiences and advice within the group:

*“And there was also a lot of inspiration from the other relatives*,* how they tackled it. And there were some who said that they actually didn’t talk about the things that were talked about over here [at the clinic]. (…) Oh God*,* can you do that? Because it’s one of the things I’ve been really bad at*,* it’s setting boundaries in relation to that*,* right? I’ve just*,* whether it was 8 o’clock at night*,* 7 in the morning*,* anytime*,* I was available. But it just wasn’t good for any of us.”*

Line, spouse, age 35.

In this quote, Line appears to have had high expectations of herself as a caregiver and consequently did not set any boundaries regarding her availability, which was exhausting for her. The challenges of setting boundaries could also be due to external pressure of expectations. A mother explained how she had previously felt like a bad parent when she met parents in other support groups who did everything for their adult child, whereas she had set up boundaries on what her responsibilities to her sons were.

*“I feel like a lot of parents sacrifice their entire lives for their children*,* right? And sometimes I’ve actually been sitting and feeling wrong. Because my attitude is*,* and I can also feel it’s hard to say now*,* but my attitude is that they’re adults [her children]. In that way*,* they’re not my responsibility anymore. I will help. I will if they reach out*,* and I will point them in a direction if they ask for it. But it’s them*,* and it’s their responsibility.”*

Tanja, mother, age 60.

Setting up boundaries was repeatedly described as very difficult, and some of the participants would have wished that this topic had been covered more thoroughly in the teaching.

In the following quote, a participant expresses that he did not think there was enough focus on the needs and boundaries of the relatives. He referred to a comment that one of the other participants made during the psychoeducation, which he could relate to:

*“She said: “But can’t I also set boundaries*,* or can’t I also have expectations for my partner?” (…) And I could hear in her voice*,* and see in her expression*,* that it carried the same feeling I had*,* which was: “Okay*,* all of this is fine*,* I can help in this way*,* but what about me? I mean*,* what about how I’m supposed to survive in these situations?”*

Morten, partner, age 33.

In the “*what about me*” statement, he appears to feel the need to defend his right to have needs and set boundaries as a relative, when he felt that the teaching did not.

In the comments from the questionnaire, the word “boundaries” was not mentioned, but it was a recurrent request (11/70) to have more focus on “the role of the relative”. This was often mentioned in relation to how to handle difficult situations, which also relates to theme E.

### Theme E: Becoming a diplomat

A participant explained how it was useful and new to him to think in terms of diplomacy, which the psychoeducation inspired him to do:

*"Something I’ve actually found really useful is the whole idea of being diplomatic. (…) Instead of thinking*,* ‘this crap*,* I can’t handle it right now because it’s going to go wrong.’ Now it’s more like*,* ‘okay*,* what can I say right now that will actually work?’ And just having that mindset about the situation*,* well*,* it helps enormously.”*

*Anders*,* partner*,* age 22*.

This quote inspired the naming of the theme: ‘Becoming a Diplomat.’ The participants emphasized the importance of diplomacy and timing in various contexts: how to address a conflict, when to react to symptoms related to BD, and when to make agreements. The question of timing seemed specifically important concerning BD as it is changeable by nature, and in certain situations, you could beneficially use this circumstance. As mentioned in theme A, when the relatives understand and recognize the course of BD, it enables them to practice “watchful waiting” and see if symptoms ease with time, instead of feeling obligated to actively handle everything. In this following quote, a father thought back on a situation where his son had shown manic symptoms and had made unreasonable plans. He figured that with the knowledge he now had from psychoeducation, he would have handled the situation differently:

*“And later on*,* I realized with the knowledge we have today*,* I should have just said back then: ‘That sounds really exciting. We can look into that when we get back from vacation.’ (…) Because a week*,* 14 days later*,* then… 3 days later*,* he would have forgotten about it. He would have moved on. So why get into conflict over something like that…"*.

*Lasse*,* father*,* age 58*.

The same ideas of ‘watchful waiting’ and ‘choosing your battles’ were also mentioned by other participants concerning when to set requirements or go into discussions.

Another parent talked about how he after the psychoeducation planned to use timing in a very concrete way: to make agreements in a ‘time of peace’ (when she was euthymic) with his daughter on how he could best help her next time she would develop depressive symptoms:

*“(…) I think in the future*,* it’s much easier. Maybe not much easier*,* but it’s easier because I understand things*,* right? I also understand what to say to her if she gets depressed. And that’s the agreement*,* some agreements*,* we can make in peacetime (…).”*

Bent, father, age 62.

A recurring request in both the interviews and written comments was for a more explicit focus on the role of the relative and strategies for handling difficult situations and conflicts. As Mads explains in his interview, he felt that the same issues kept arising between him and his wife, and he still lacked the ‘diplomatic tools’ to navigate these challenges.

*(…) now we’re getting to this issue*,* and I can see how it’s going to play out. I just don’t really have the tools or anything to step in and say: “well*,* let’s talk about how we can*,* talk about it in a different way”*.

*Mads*,* spouse 31*.

As mentioned in theme A, half of the written comments in the questionnaire requested more time for group discussions often with the argument that it would enhance peer-to-peer knowledge sharing. This could in turn help ensure a greater focus on the role of relatives. Similarly, a guest teacher with a caregiver background was suggested to further support this knowledge sharing:

*“I would encourage including a lecture by a relative in the teaching. It would be very valuable to hear their perspectives*,* for example*,* on how different situations are handled.”*

*Written commentary from a wife age 37*.

## Discussion

This study aimed to *get insights into the relatives’ experience of the psychoeducation program*,* how it influenced them*,* and identify the factors that appeared to contribute to these effects and experiences.* Through our analysis, we arrived at the following themes: (A) Relating through shared experiences, (B) Understanding the land of Bipolar Disorder, (C) Trusting the Treatment Means Sharing the Burden, (D) Setting boundaries to protect Yourself, and (E) Becoming a Diplomat.

Based on our themes we can roughly summarize how the group-based psychoeducation worked -when it worked: When a relative got the right prerequisites in terms of knowledge about the illness, treatment, and psychiatric system (themes B and C), as well as the opportunity to relate to others in similar situations (theme A), it enables a positive and constructive role as a relative, where the relative can handle complex and difficult situations diplomatically (E) while also taking care of oneself (D).

However, multiple factors moderated the positive effects of the psychoeducation, which we will discuss in the following.

What surprised us the most was that while the large group was generally accepted by the participants, they consistently requested to be matched with relatives in *more* similar situations. They emphasized that simply being a relative of a newly diagnosed BD patient was not specific enough. The type of relationship—whether they were a partner or a parent—was highlighted as the most important factor, followed by distinctions such as BD type 1 versus type 2 and whether the relative was in a crisis or managing everyday life. The question of ‘a good match’ was a determining factor in terms of whether they felt they could relate to the others (Theme A) and whether the education suited their needs (Theme B). Some of the participants felt that their knowledge had greatly improved during the psychoeducation, and others (especially partners) said that they already knew large parts of the course content. The latter group expressed that they would have preferred *less* focus on factual knowledge about BD and *more* emphasis on the role of the caregiver. They specifically requested focus on how to set boundaries (Theme D) and how to handle difficult situations (Theme E). This request was often accompanied by a wish for more group discussions, as these could enhance peer-to-peer knowledge sharing on boundary-setting and conflict resolution.

The different needs and expectations reflect that the group represented relatives in diverse situations and on different “stages” in their learning process [33]. A phenomenological study by van den Heuvel et al. identified four stages in the learning process of informal caregivers, based on their experience of caring for someone with BD (no psychoeducational intervention was involved in this study). The first stage was to seek information to understand BD, the second stage was overcoming the dilemmas stemming from living with someone with BD, the third was to understand the negative consequences of emotional overinvolvement and solving this by the fourth stage which was a division of responsibilities. Finally, the fifth stage was about defining and delimiting their own personal supporting role to enhance self-management in the person with BD. The last two stages are very similar to our two last themes: Setting boundaries (D) and Becoming a Diplomat (E).

We discovered that although the Copenhagen Affective Disorder Clinic is a recognized, highly specialized clinic with good treatment outcomes [17, 34], this was not necessarily something the relatives were aware of. On the contrary, some relatives had been burdened by concerns about the quality of the treatment and felt a responsibility for it that they did not know how to handle. The lack of prior knowledge of the treatment and clinic is extra notable here, as we interviewed relatives who we assume are particularly resourceful and proactive, as they attended the sessions and volunteered for the interview. In Theme C, we describe how the education relieved them, as they could now trust the treatment and thereby shift some of the burden from their own shoulders to the clinic. In this context, it was particularly beneficial that the psychoeducation was delivered in-house at the clinic by experienced clinicians.

The support needs of informal caregivers for persons with BD were explored in a recent qualitative study by Speirs et al. [33]. They concluded that informal caregivers needed various types of support, including information on BD and its treatment, opportunities to share experiences with other carers, a sense of inclusion from health professionals, as well as coping strategies and psychological skills: managing stress, responding to different situations, practicing self-care, and enforcing boundaries. These support needs closely align with our findings, which suggests that the group-based psychoeducation intervention at the Copenhagen Affective Disorder Clinic has the potential to address core caregiver needs.

Our themes and the roughly outlined mechanisms of action they present also align well Lazarus and Folkman’s “stress-appraisal-coping” model [35]. This model has previously been used to describe the effects of psychoeducation for relatives in the following way: By increasing the relatives’ knowledge of BD, psychoeducation can change their appraisal of the situation, and potentially foster positive coping strategies such as self-care, problem-solving, and good communication skills [10, 18, 36, 37]. The positive coping strategies mentioned here are almost identical to themes D and E in our analysis: Setting boundaries to protect yourself (D) and Becoming a diplomat (E). When combined, these two themes form the basis of a compelling “caregiver role model*”*, which encompasses all five themes: the *‘Diplomat with Boundaries*.’

The concept of a diplomat is fitting, as the skills associated with diplomacy closely mirror the challenges faced by relatives. Diplomats must have alliances (Theme A) and a good understanding of the foreign country in which they operate (theme B and C). Also, diplomats must defend their own country’s interests (Theme D) while demonstrating good communication and conflict management skills—hallmarks of good diplomacy (theme E).

### Implications for clinical practice

We will highlight the following two points addressing both (a) the practical organization of psychoeducation and (b) a suggestion for building psychoeducation around the concept of a “diplomat with boundaries”.

A) The participants appreciated the group-based format of the psychoeducation. The relatively big groups were feasible, but it could be considered to give more time for discussions between participants in smaller groups, preferably in groups with relatives in similar situations based on their relationship type and the type of BD. Additionally, offering psychoeducation to relatives at the start of the patient’s treatment at the clinic could better align them in terms of their learning stage, making their needs more similar.
B) Our participants expressed that it was difficult to find out how to be a “good caregiver” while also taking care of themselves and hoped that the psychoeducation could help guide them. A possibility could be to use the concept of “diplomats with boundaries” as a prevalent reference point throughout the sessions. A concept like this could both serve as a common thread through the sessions and be a useful mental picture that the relatives could build further on. Also, it would be a strength to know, that the idea of “diplomats with boundaries” was conceptualized through interviews with relatives, and thus indirectly a helping hand between peers.

### Strengths and limitations of the study

The participants knew that JRS is a medical doctor employed in the Copenhagen Affective Disorder research Center (CADIC; https://www.psykiatri-regionh.dk/cadic) and thereby connected to the clinic which offers the psychoeducation program. This could maybe influence their responses in the qualitative interviews, as they might adapt their thoughts on the subject out of politeness, and respect, or to align with what they expected would resonate with JRS and/or the RCT. JRS tried to address this potential problem by creating a relaxed atmosphere and emphasizing that their honest opinion and experiences were important.

Regarding transferability, it should be noted that all patients at the Copenhagen Affective Disorder Clinic have been diagnosed with BD within the last two years. Although some of the patients have had symptoms for several years before receiving the bipolar diagnosis, the fact that many patients are in the early stages of their illness may mean that the effect of psychoeducation is more significant than if the patient group had been more ‘chronic’ [38]. This is not an actual limitation of the study, but rather a consideration regarding the transferability of the results to other groups. It is a strength that we were also able to include the written free-text comments from the questionnaire of the parallel RCT, which confirmed our analysis.

## Conclusions

Group-based psychoeducation for relatives of patients with BD in relatively large groups was a feasible format, but the analysis identified factors that could be modified to enhance the impact. Participants emphasized that parents and partners differed in prior knowledge and their expectations of psychoeducation. Thus, within the setting of large psychoeducation groups such as those in the Copenhagen Affective Disorder Clinic, participants could be matched in group discussions by the type of relation with the patient and possibly also the type of BD. Our participants highlighted how difficult it was to find out how to be a “good caregiver” while also taking care of themselves. We propose that the group-based psychoeducation be based on the concept of ‘diplomat with boundaries’, which is a positive model for caregiving that was conceptualized through the analysis. A relative who is a ‘diplomat with boundaries’ is characterized by having knowledge about BD, good communication skills, and strategic conflict management. The addition “… with boundaries” refers to being able to set up healthy boundaries in the relationship. Group-based psychoeducation can help the relatives in the direction of becoming good ‘diplomats with boundaries’.

## Supplementary Information

Supplementary Material 1.

Supplementary Material 2.

## Data Availability

The data analyzed in this study can be made available from first author JRS on reasonable request.

## Abbreviations

BD: Bipolar Disorder
TA: Thematic Analysis
RCT: Randomized Controlled Trial
CADIC: Copenhagen Affective Disorder research Center
