# Supplementary material for: Becoming diplomats with boundaries - a thematic analysis of relatives’ experiences with group-based psychoeducation about bipolar disorder
Source: BMC Psychiatry. 2025 Sep 1;25:843. doi: 10.1186/s12888-025-07219-y (PMC12400627; doi:10.1186/s12888-025-07219-y)
Supplement: Supplementary file 1 — Supplementary Material 1. [file 12888_2025_7219_MOESM1_ESM.docx]

Appendix 1: Interview guide translated from Danish to English

**At the beginning of the recording say:**

*Today is XX.XX.XX. It's JRS speaking right now, and with me, I have my interview participant XX, ID number XX.*

*Before we start, I'd like to let you know that we have 1 hour for the interview.*

*I have a list of questions that I'd like to go through with you. Along the way, I'll ask about your answers and have you elaborate on them, and sometimes it may seem a bit strange. That's because I need to make sure I understand what you mean by your words, not what I think you mean.*

**Introduction:**

How have you experienced being a relative since XX was diagnosed and has been treated at this department?

Why did you sign up for the relatives' course?

What were your expectations for it – what did you hope to get out of it?

**Main questions:**

What significance has it had for you to attend the course?

- How has it helped you? (Questions about stress, burden, etc.)

- What do you think is the reason for *any change in stress level, etc.*?

- How have you been able to use the knowledge you gained in the course? Can you provide some concrete examples?

- Has it made a difference regarding loneliness/doubt/shame/experience of stigma?

What significance has it had on your and your relative's relationship?

- What significant knowledge have you gained about your relative?

- What has that knowledge meant for your experience of being able to help?

- What has that knowledge meant for your relationship and your thoughts about the future?

Do you think that attending the course will change how much you, as a relative, will be involved in XX's treatment in the future?

**Closing:**

Is there anything you would like to change about the course? Anything you felt was missing or something you found unnecessary?
