# Supplementary material for: Becoming diplomats with boundaries - a thematic analysis of relatives’ experiences with group-based psychoeducation about bipolar disorder
Source: BMC Psychiatry. 2025 Sep 1;25:843. doi: 10.1186/s12888-025-07219-y (PMC12400627; doi:10.1186/s12888-025-07219-y)
Supplement: Supplementary file 2 — Supplementary Material 2. [file 12888_2025_7219_MOESM2_ESM.docx]

Informed consent ID no.__________________

**Consent regarding participation in qualitative interview in connection with the research project R-bipolar.**

Name: __________________________________

Date of Birth: ____________________________

I hereby consent to participate in the interview of the study and understand that it is anonymous and voluntary to participate in the study, and that I can withdraw my consent at any time. All information given during the interview is completely confidential. I am aware that the interview will be recorded. The audio recording will be deleted once the analysis is completed, and at the latest within 5 years.

Participating in the interview, or withdrawing my consent, will have no consequences for my current or future treatment.

I have received oral and written information about the study and have been given a copy of this consent form for my own use.

Date:

Signature:

### Appendix 2: Consent form translated from Danish to English
